# Multidrug resistant bacteria isolated from nosocomial infections at University Teaching Hospital of Point-G, Bamako, Mali

**Published:** 2023-01-31

**Authors:** Maiga Aminata, Beye Seydina Alioune, Cissoko Yacouba, Dicko Oumar Agaly, Diarra Bassirou, Traoré Abdoulaye, Coulibaly Djibril Mamadou, Koné Drissa, Diarra Lobogal, Coulibaly Djaminatou, Coulibaly Youssouf, Maiga Ibrahim Izetiégouma, Fofana Djeneba Bocar

**Affiliations:** 1 Medical Biology and Hospital Hygiene Laboratory, Point-G University Hospital, Bamako, Mali.; 2 Faculty of Medicine and Odontostomatology, University Teaching Hospital of Point-G, Bamako, Mali.; 3 Point-G University Hospital Resuscitation Unit, Bamako, Mali.; 4 Department of Infectious and Tropical Diseases, Point-G University Hospital, Bamako, Mali.; 5 University Center for Clinical Research, Point-G University Hospital, Bamako, Mali.; 6 Faculty of Pharmacy, Point-G University Hospital, Bamako, Mali.; 7 National Institute for Training in Health Sciences, Mali.

**Keywords:** Nosocomial infections, multi-resistant bacteria, UTH of Point-G

## Abstract

An infection is said to be nosocomial or hospital if it is absent when the patient enters the hospital and it appears and develops at least 48 h late. The objective of this study was to determine the resistance phenotypes of bacteria isolated from nosocomial infections at the University Teaching Hospital of Point G. Urine, blood, pus, skin and bronchoalveolar fluid samples were taken in different units, and bacteria isolations were performed on usual selective media such as Drigalski Colombia agar supplemented with nalidixic acid and colistin and 5% sheep blood and chocolate agar. Identifications of bacteria such as *Enterobacteriaceae, Pseudomonas and acinetobacter, and* Staphylococci were done using API20^E^ gallery, API20^NE^ gallery and catalase/oxidase tests, and the Pastorex Staph kit respectively. The antimicrobial susceptibility testing was performed on Mueller-Hinton agar using the diffusion method. A total of 463 patients were inpatients for at least 48 h in the different units, and a nosocomial infection was notified in at least 57 patients (12.3%). A total of 65 episodes of nosocomial infections were observed in these 57 patients. Of the bacteria isolated, multidrug-resistant bacteria (MDR) represented 63.7% (n=36). These were extended-spectrum beta-lactamase (ESBL)-secreting *Enterobacteriaceae* (n=21), high-level cephalosporinase (n=13) and methicillin-resistant coagulase-negative Staphylococci (n=2). Despite this high number of multi-resistant bacteria isolated in this study; colistin and amikacin had very good activity on *enterobacteriaceae.* The results show the need to strengthen hygiene in the intensive care units in order to fight against nosocomial infections at the UTH of Point G.

## INTRODUCTION

An infection is said to be nosocomial or hospital if it is absent when the patient enters the hospital and it appears and develops at least 48 h late. According to the World Health Organization (WHO), more than 1.4 million people worldwide suffer from hospital-acquired infections (OMS, 2017). In the United States, the risk of being a victim of this type of infection has increased steadily over the past decades and resulted in expenditure estimated at $4.5 to 5.7 billion per year (Statistiques, 2017). In France, 6 to 7% of hospitalizations are complicated by a more or less serious NI. The rates of healthcare-associated infections (HAIs) and bacterial resistance in developing countries are 3 to 5 times higher than international standards. In countries with limited resources, such as West African nations, other features, more specifically socioeconomic and behavioral factors, contribute to exacerbate this problem (Ouedraogo et al., 2017). The nosocomial infections fall within a range of colossal costs going from around 340 euros for a urinary tract infection to 4000 euros for an infection contracted in the intensive care unit (Boulahouat and Aliziane, 2019). HAIs can extend the length of stay to 10 days, increase costs (US$5,000 to US$12,000) and mortality (by a factor of 2 to 3) (Rosenthal, 2008; Boulahouat and Aliziane, 2019).

Resource-limited countries have higher rates of device-associated healthcare-associated infections (HAIs), including central line-associated bloodstream infection (CLAB), ventilator-associated pneumonia (VAP), and infection Catheter-Associated Urinary Tract (CAUTI), 3 to 5 times higher than rates reported in intensive care units in North America, Western Europe and Australia (Rosenthal, 2008). Africa has the highest rate estimated at 25% (Kakupa et al., 2016). In Côte d’Ivoire, the emergence of multidrud-resistant bacteria (MDR) responsible for infections in hospitals has been observed in recent years (Guessend, 2008; Méité et al., 2009) and in Senegal, global incidence multi-resistant bacteria was 5.5% with an incidence density of 5 cases per 1000 patient-days at the University Hospital of Fann in Dakar (Fortes et al., 2015).

In Mali, several studies have been carried out to determine the extent of NI, and different prevalence rates varying was found from 4.72 to 29. 4% (Dembélé, 2015; Dembele, 2017; Beye et al., 2022; Bocoum et al., 2020; Dicko et al., 2022).

Moreover, a study conducted in 2002 in five hospitals in Bamako showed a prevalence of NI at 14.4% (Maiga, 2002) Thus, the objective of this study was to determine the resistance phenotypes of bacteria isolated from nosocomial infections at the University teaching Hospital of Point G.

## METHODS

### Study design and settings

This was a descriptive and analytical cross-sectional prospective study over a period of 6 weeks from 1^st^ July to 18^th^ August, 2019 which was carried out in 10 departments of the University Teaching Hospital (UTH) of Point G including the Intensive Care Unit, general surgery A and B, gynecology-obstetrics, neurology, nephrology and hemodialysis, rheumatology, internal medicine, infectious and tropical diseases; and urology. The University Teaching Hospital of the Point G is the third-pyramidal’ reference in Mali, and has 522 beds divided between the surgical, intensive care and medical departments.

All hospitalized patients were included in this study for at least 48 h with a suspicion of infection or confirmed by the presence of at least two criteria of the systemic inflammatory response syndrome (SIRS) such as a temperature >38.5°C or < 36°C, heart rate > 90 beats per minute, respiratory rate > 20 cycles/minute or PaCO_2_ < 32 mm Hg, white blood cells > 12000/mm^3^ or < 4000/mm^3^ or > 10% of immature forms, observed after at least 48 hours of hospitalization, and which were neither present nor incubating at the time of hospitalization.

### Sample types and patients

The samples were blood cultures which were taken by nurses or interns at the time of fever peaks (temperature ≥ 38.5°C) or in the event of hypothermia (temperature < 36°C) by puncture of a non-perfused vein in two different bottles (aerobic, anaerobic). The cyto-bacteriological examinations were carried out on the first urines in the morning, or after clamped the urinary catheter for about 20 minutes. The suppuration samples were collected under strict aseptic conditions using a swab moistened with sterile 0.9% saline solution, and Broncho alveolar lavage (BAL) was performed by a pulmonologist using a bronchoscope after instillation of 100 mL of sterile isotonic saline through the trachea, before the secretions were aspirated and collected in a sterile bottle. All samples were taken according to the site of infection and sent directly to the laboratory for further investigation.

### Laboratory procedures

#### Isolation of bacterial strains

Bacterial cultures were carried out on the usual selective media such as Drigalski (Bio-Rad, France) Colombia agar supplemented with nalidixic acid and colistin and 5% sheep blood and chocolate agar (Bio-Mérieux, France). While Enterobacteriaceae were identified using the API20^E^ gallery (Bio-Mérieux, France), *Pseudomonas* and *Acinetobacter* were identified using the combination of catalase/ Oxidase (Bio-Rad, France), and the API 20^NE^ gallery (Bio-Mérieux, France). *Staphylococcus* was identified using the Pastorex Staph kit (Bio-Rad, France).

#### Antimicrobial susceptibility testing and reading

The antibiogram was carried out on Mueller-Hinton agar (Bio-Rad, France) using the diffusion technique in agar medium. After inoculation and drying of the Mueller-Hinton agars, the discs of blotting paper impregnated with the following antibiotics were tested for Gram-negative bacilli: ticarcillin (30μg), nalidixic acid (30μg), ciprofloxacin (5μg), cefotaxime (30μg), amoxicillin + clavulanic acid (20/10μg), ceftazidime (30μg), cefoxitin (30μg), imipenem (10μg), chloramphenicol (30μg), tetracycline (30μg), gentamicin (15μg), amikacin (30μg),sulfamethoxazole (30μg), trimethoprim (30μg), colistin (30μg), by depositing them on the surface of the inoculated dishes using a disc dispenser (Bio -Rad, France). All strains were systematically detected for the production of an extended-spectrum beta-lactamase by the synergy test. This test consists of adding to the disc of amoxicillin associated with clavulanic acid and those of third generation cephalosporins. Then, after incubation at 37°C for 18 to 24 h, the production of broad-spectrum beta-lactamase results in the appearance of an image of synergy or champagne cork between the discs. Before being used, an internal control of the antibiotic discs was carried out using the reference strain of *Escherichia coli* ATCC 25922 to ensure the validity of the results obtained (CA-SFM, 2019). The antibiotic discs for which the inhibition diameters obtained are within the interval of the critical diameters present in the reading chart of the CA-SFM, 2019 were considered to be compliant.

Similarly, the blotting paper discs impregnated with the following antibiotics were tested on the strains of Staphylococci: penicillin G (10UI), amoxicillin (10μg), cefoxitin (30μg), oxacillin (5μg), chloramphenicol (30μg), kanamycin (30 IU), gentamicin (15μg), tobramycin (10μg), netilmicin (30μg), amikacin (30μg), tetracycline (30μg), ciprofloxacin (5μg), fusidic acid (30μg), fosfomycin (50μg), sulfamethoxazole (30μg), trimethoprim (5μg), erythromycin (15μg), lincomicin (15μg), pristinamycin (15μg).

In addition, strains of Enterobacteriaceae and *Acinetobacter* resistant to amoxicillin (30μg), ticarcillin (30μg), amoxicillin+clavulanic acid (20/10μg), cefalotin (30μg), cefoxitin (30μg), ceftazidime (30μg) and cefotaxime (30μg) but sensitive to imipenem (10μg) were considered high-level cephalosporinase producers (Courvalin and Philippon, 1989).

Enterobacteriaceae strains that have a positive synergy test between amoxicillin + clavulanic acid and 3rd generation cephalosporins (cefotaxime and ceftazidime) were considered as extended-spectrum beta-lactamase producers (Bonnet, 2012). Moreover, the strains of coagulase-negative Staphylococci resistant to cefoxitin and oxacillin were considered resistant to methicillin (Carttoir and Leclerq, 2012).

The readings of the results were performed by measuring the diameters of inhibition using a caliper or a graduated ruler. The results were transcribed into sensitive (S), intermediate (I), or resistant (R) categories according to the recommendations of the CA-SFM-EUCAST, 2019 and the different phenotypes of the strains were determined according to the families of antibiotics tested.

### Statistical and data analysis

The data were collected on a pre-established form from admission and treatment registers, temperature sheets, medical records, surgery report registers, intensive care unit reporting sheets, results of bacteriological tests. A delegate has been appointed to inform the investigator in the event of suspected infection in each department concerned.

Data were entered and then analyzed using SPSS version 22 software. Quantitative variables were expressed as the mean (± standard deviation). Qualitative variables were expressed as a proportion.

### Ethical statement

The leadership of the UTH of Point G was informed before starting the study, and study was approved by the ethics committee of the Faculty of Medicine of Dentistry under registration number of n°2019 /76/EC/FMOS.

## RESULTS

### Global frequency of nosocomial infections

A total of 463 patients were inpatients for at least 48 h in the different departments surveyed and 57 patients (12.3%) had at least one nosocomial infection. We observed 65 episodes of nosocomial infections in the 57 infected patients.

Forty-nine patients had one episode and eight had two episodes, including six patients with two episodes of urinary tract infection and two patients with two episodes of surgical site infection.

**Figure 1.**
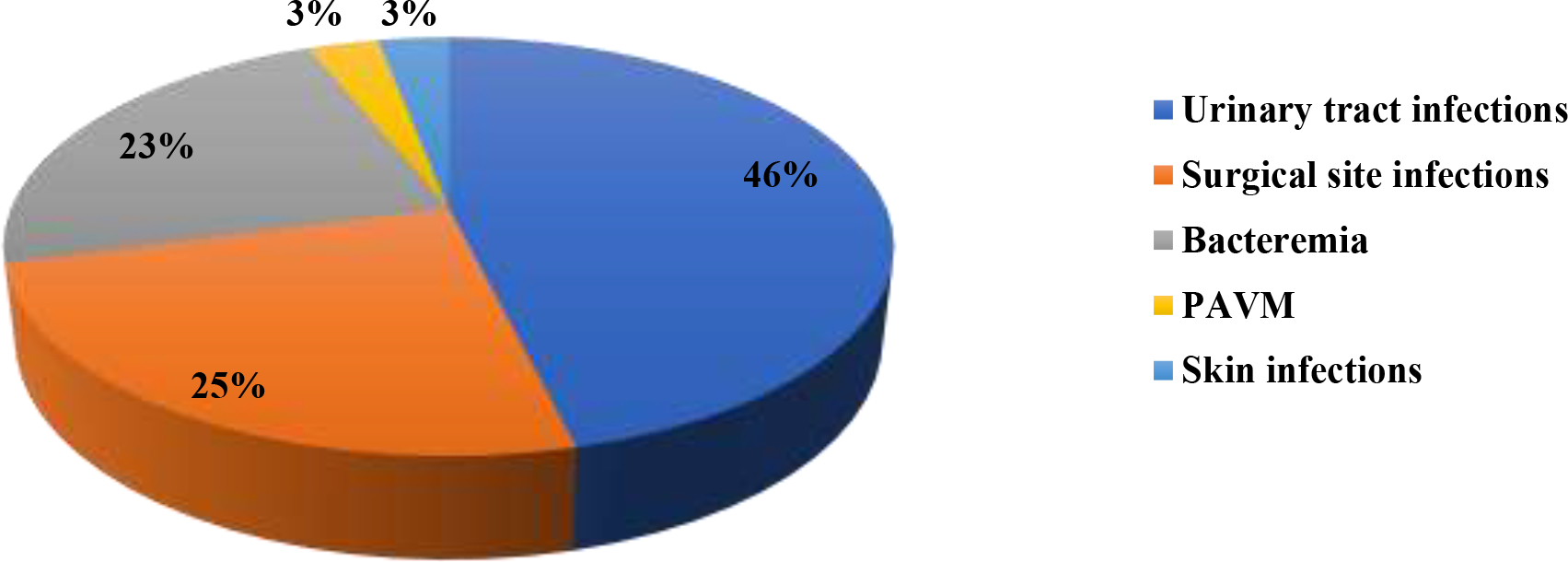
The different nosocomial infections. PAMV = pneumonia acquired under mechanical ventilation. Source: Authors

Of the different nosocomial infections, 30/65 (46.1%) were urinary infections, 16/65 (24.6%) were surgical site infections, 15/65 (23.1%) were bacteremia, 2/65 (3.1%) were skin infections and 2/65 (3.1%) were pneumonia acquired under mechanical ventilation (PAMV) (Figure 1).

### Bacterial strains isolated and resistance pattern

The main microorganisms isolated were Enterobacteriaceae, mainly *E. coli* and *K. pneumoniae*. *E. coli* was the most isolated germ in urinary tract infections, in surgical site infections and in cutaneous infections. *Acinetobacter baumannii* was the most microorganisms isolated in bacteremia, and *Citrobacter freundii* and *Pseudomonas putida* were the germs isolated in PAMV.

**Figure 2.**
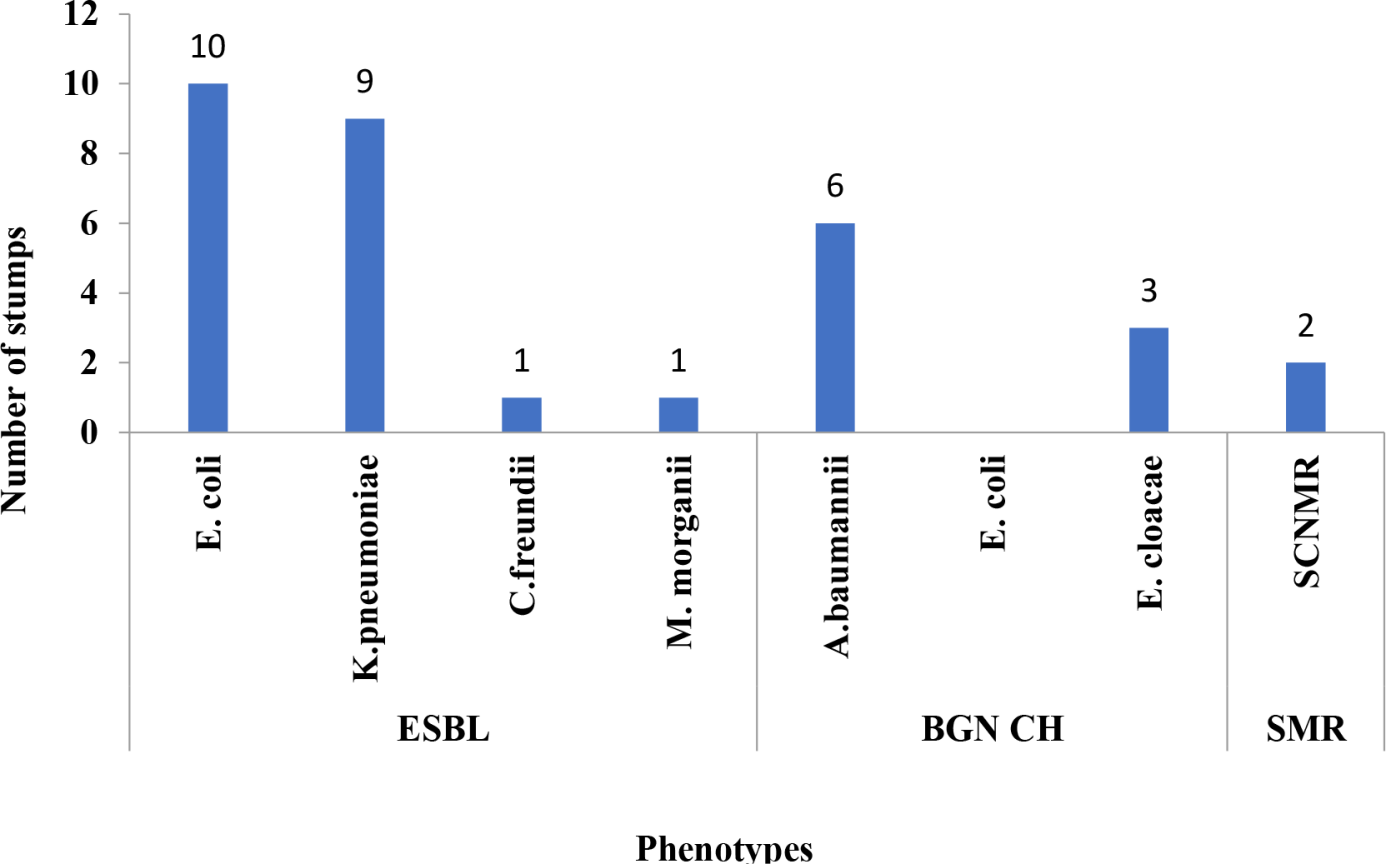
Distribution of multi-resistant bacteria responsible for nosocomial infections. ESBL = extended-spectrum beta-lactamase, BGNCH = Gram-negative bacilli Cephalosporinase hyperproduced, SCNMR= Coagulase Negative Methicillin Resistant *Staphylococcus.* Source: Authors

Multi-resistant bacteria (MDR) at 63.2% (n=36) were the micro-organisms found in all patients. These were ESBL-secreting *Enterobacteriaceae* (n=21), high-level cephalosporinase (n=13) and methicillin-resistant coagulase-negative Staphylococci (n=2) (Figure 2).

The number of resistant strains corresponds to the summary of the intermediate and antibiotic-resistant strains (Table 1).

## DISCUSSION

The sample was exhaustive but there are limits and difficulties such as poorly informed hospitalization records, certain additional examinations were not carried out due to lack of financial means. There could be the possibility of selection bias among patients in the infectious and tropical diseases department where patients were admitted with suspicion of fever. In this study, 54 microorganisms were isolated, Enterobacteriaceae were more frequent (61.1%), followed by non-fermenting Gram-negative bacilli (22.2%) and Gram-positive cocci (11.1%). Our results were consistent with literature data (Pilly, 2015). In our study, the micro-organisms were more isolated in the urine (42.6%), then in parietal suppuration (25.9%) and bacteremia (14.8%), then in skin infections (7.4 %) and PAMV (3.7%). These results confirm those of the national survey on the prevalence of nosocomial infections and healthcare-associated infections in France in 2017 (Daniau et al., 2018), of Issa Maigardie in Mali (Maigardie, 2007) and of Déguénonvo et al. (2015) at the CHU Fann in Dakar in 2015 (Déguénonvo et al., 2015). However, Keita et al. (2016) in Conakry reported rates of urinary tract infections lower than ours (16.1%) (Keita et al., 2016) and they were the second cause of nosocomial infections in their studies. An outline of an epidemic of *E. coli* infection of the surgical site was observed in the general surgery department B in two patients: the two strains of *E. coli* had the same antibiotic type, as well as a sort of cluster of urinary tract infection to *Acinetobacter baumannii* was observed in the urology department in two patients, the two strains had the same antibiotic type. Such clusters can be explained by hand-borne contamination due to poor hand hygiene in these departments. Such epidemics had not been described at the Point-G University Hospital.

Bacterial resistance to antibiotics has been considered since 2014 by the WHO as a public health priority (Durand et al., 2016). In this study, the frequency of multi-resistant bacteria (MDR) was very high (66.7%) unlike other studies which are between 10.3 to 32.9% in the USA depending on the center, 13% in Europe, and 29.26% in the Maghreb (Harris et al., 2013; Derde et al, 2014; Bourich, 2011). However, in Africa the very often irrational use of antibiotics may explain this high rate. Moreover, in intensive care, as in the majority of long-stay hospitalization units, the prescription of antibiotics is very high, all the studies convergent recognize a decisive role for prior antibiotic therapy as a major factor in the appearance of hospital flora with resistant bacteria: either antibiotic therapy for more than 24 h in the previous days, or less recent but prolonged antibiotic therapy (BMR-Raisin, 2016; Diakity, 2010). Among the MDR, we isolated the broad-spectrum beta-lactamase-secreting Enterobacteriaceae (ESBL), the main species of which were *Escherichia coli* and *Klebsiella pneumoniae*. Bacteria secreting hyper-produced cephalosporinase were *Acinetobacter baumannii*, *Enterobacter cloacae* and *E. coli*. Methicillin-resistant coagulase-negative staphylococci (Figure 2). Maigardie in 2007 in Mali (Maigardie, 2007), Keita et al in Guinea in 2016 (Keita, 2016), Durand A. et al. in France observed the same results as us. Similar results were also observed in other studies in 2016 (Durand et al., 2016). All of our strains of *E. coli, K. pneumoniae* were sensitive to amikacin, (13/16), colistin and (10/16). The sensitivities of *E. coli* for these antibiotics have been constant in Mali since 2010 as shown by the studies of Zitti and Diakité (Diakity, 2010; Ziti, 2014). All of our *E. cloacae* strains were sensitive to colistin and amikacin in this work.

The strains of *Acinetobacter baumannii* found were all multiresistant but were sensitive to colistin and amikacin. This activity was comparable to which found by Diakité with 100% activity for each molecule (Pellegrino et al., 2002).

Ticarcillin, imipenem, ceftazidim, gentamicin, amikacin and ciprofloxacin had very good activity on all strains of *Pseudomonas* as reported by Diakité (Diakity, 2010) in his work with similar results for all these molecules except for ciprofloxacin where the activity was borderline (66.7%) and ceftazidim (33.3%) for which the activity was less. Zitti (2014) in his study had reported a limited activity of ceftazidim (53.3%) and a lower activity of amikacin (46.7%), gentamicin (40%) and ciprofloxacin (20%) on strains of *P. aeruginosa*. Indeed, *Pseudomonas* is bacteria that combine many mechanisms of resistance to antibiotics, requiring regular analysis of their activity. In addition, they are implicated in several cases of nosocomial infections, particularly in intensive care units (Giuliani et al., 2005).

### Conclusion

This study reported that colistin and amikacin still retain very good activity on all strains of Enterobacteriaceae in healthcare settings despite a high level of multi-resistant bacteria. These data show the need for infection control interventions in Mali and a rigorous and effective application of disinfection procedures and better hospital hygiene as well as the rational use of antibiotics. These measures will enable effective infection control and monitoring of these interventions as an indicator of quality of care.

## Figures and Tables

**Table 1.** Resistance to antibiotics of the different peces of ESBL.

Antibiotics	Resistance of strains (I+R)		
	*E. co li N*= 16	*K. pneumonia N*= 10	*E. cloacae N*= 4

**Beta-lactams**			
Amoxicillin	16	10	4
Amoxicillin + aclavulanic acid	16	10	4
Ticarcillin	16	10	4
Cefalotin	16	10	4
Cefotaxim	16	10	4
Ceftazidim	16	10	4
Cefoxitin	14	9	4
Imipenem	1	10	0
**Aminosides**			
Gentamicin	16	10	4
Amikacin	16	10	4
**Quinolones**			
Nalidixic acid	16	8	3
Ciprofloxacin	16	9	4
**Other antibiotics**			
Tetracyclin	13	7	4
Colistin	14	0	4
Chloramphenicol	14	6	3
Sulfonamides	12	3	4
Trimethoprim	11	3	3

Colistin, imipenem and amikacin were the most active antibiotics against *Escherichia coli, Klebsiella pneumoniae, Acinetobacter baumannii* and *Enterobacter cloacae. Pseudomonas* strains were sensitive to: ceftazidime, imipenem, gentamicin, amikacin and ciprofloxacin. The only strain of *Citrobacter freundii* isolated was resistant to all the molecules tested and *Morganella morganii* was sensitive only to amikacin.

Source: Authors
